# *CDKN2B-AS1* polymorphism rs1333049 is associated with advanced carotid artery atherosclerosis in a Slovenian population

**DOI:** 10.17305/bb.2024.10894

**Published:** 2024-09-19

**Authors:** Jernej Letonja, David Petrovič, Danijel Petrovič

**Affiliations:** 1 Laboratory for Histology and Genetics of Atherosclerosis and Microvascular Diseases, Institute of Histology and Embryology, Faculty of Medicine, University of Ljubljana, Ljubljana, Slovenia; 2 Institute of Histology and Embryology, Faculty of Medicine, University of Ljubljana, Ljubljana, Slovenia

**Keywords:** *CDKN2B-AS1*, rs1333049, atherosclerosis, advanced carotid atherosclerosis, polymorphism

## Abstract

Several studies have reported an association between the 9p21 region in the human genome and atherosclerosis. The rs1333049 polymorphism is a single nucleotide polymorphism (SNP) in *CDKN2B-AS1*, located in the 9p21 region. The aim of our study was to investigate the association between the rs1333049 polymorphism and advanced carotid atherosclerosis, as well as its effect on *CDKN2B* expression in endarterectomy sequesters. In our case-control study, we included 881 participants, divided into two groups. The case group comprised 308 participants with advanced atherosclerosis of the internal or common carotid artery (CCA) (stenosis > 75%) who underwent a revascularization procedure. The control group included 573 participants without hemodynamically significant carotid atherosclerosis. We analyzed the rs1333049 polymorphism using the StepOne real-time polymerase chain reaction (PCR) and TaqMan SNP genotyping assay. We found a statistically significant association according to the co-dominant (*P* ═ 0.014, OR ═ 3.29, 95% CI: 1.32–8.91, and *P* ═ 0.015, OR ═ 2.50, 95% CI: 1.22–5.37) and dominant (*P* ═ 0.006, OR ═ 2.74, 95% CI: 1.36–5.71) models. We performed immunohistochemical analysis of *CDKN2B* expression on 26 endarterectomy sequesters. The C allele of rs1333049 was associated with a lower numerical area density of *CDKN2B*-positive cells in atherosclerotic plaques. In conclusion, the C allele of the rs1333049 SNP is associated with an increased risk of developing advanced carotid atherosclerosis and lower *CDKN2B* expression in the plaques.

## Introduction

Atherosclerosis is a chronic inflammatory disease characterized by the formation of atherosclerotic plaques in the arterial intima. The process begins with the accumulation of low-density lipoproteins (LDL) in the arterial intima at sites of turbulent blood flow. Macrophages and vascular smooth muscle cells (VSMCs) engulf oxidized LDL particles, transforming into foam cells. As the plaque develops, cellular debris, lipids, and calcium build up [1, 2]. Atherosclerosis can affect any artery in the body and often remains asymptomatic for years until complications arise, with symptoms depending on the affected artery [3]. Several studies have found an association between the 9p21 region of the human genome and atherosclerosis [4–6]. The gene for cyclin-dependent kinase inhibitor 2B antisense RNA 1 (*CDKN2B-AS1*) is located within the *CDKN2A/2B* gene cluster in this region. *CDKN2B-AS1* encodes a long non-coding RNA (lncRNA) that modulates the expression of *CDKN2A, CDKN2B*, and methylthioadenosine phosphorylase (*MTAP*) [6]. *CDKN2A* (p16/Ink4a) and *CDKN2B* (p15/Ink4b) inhibit cyclin-dependent kinases and are important tumor suppressors [7, 8]. These genes are downregulated in several cancers, including melanoma, leukemia, hepatocellular carcinoma, and colorectal carcinoma [7–11]. Studies in mice have shown that knocking out *CDKN2B* results in larger atherosclerotic plaques with increased lipid accumulation due to reduced efferocytosis [12]. Loss of *CDKN2A/2B* is also linked to changes in the phenotype of T cells and monocytes [13, 14]. *CDKN2A* and *CDKN2B* additionally inhibit the proliferation of VSMCs [15]. The rs1333049 polymorphism, a single nucleotide polymorphism (SNP) in the *CDKN2B-AS1* gene, affects an lncRNA. The rs1333049 variant is associated with decreased levels of *CDKN2A* and *CDKN2B* in atherosclerotic plaques and increased proliferation of VSMCs [15]. In a meta-analysis, the C allele of rs1333049 was found to increase susceptibility to ischemic stroke (IS) [16]. The aim of our study was to investigate the association of the rs1333049 polymorphism with advanced carotid atherosclerosis in a Slovenian population of Caucasian descent.

## Materials and methods

### Patients

We conducted a case-control study including 881 unrelated Slovenian participants of Caucasian descent. The participants were divided into two groups: 308 patients with advanced atherosclerosis of the internal carotid artery (ICA) (defined as >75% lumen obstruction) formed the case group, while 573 participants undergoing routine cardiovascular examination at outpatient clinics comprised the control group. The control group included participants of both sexes who had no signs of advanced atherosclerosis, as verified by ultrasound examination of the ICA or common carotid artery (CCA), or who had only mild atherosclerosis (hemodynamically insignificant stenosis <50%). The case group had all undergone revascularization procedures (stent implantation for 274 patients or endarterectomy for 34). All subjects were recruited from three Slovenian healthcare institutions (International Center for Cardiovascular Diseases MC Medicor, Izola, University Clinical Center Maribor, and General Hospital Izola) between 2010 and 2023. Exclusion criteria included stenosis of the aortic arch or right subclavian artery, carotid artery stenosis of non-atherosclerotic origin, presence of a neck tumor, or incomplete data. Vascular ultrasound examinations were used to assess the degree of stenosis. In some patients, computed tomography (CT) angiography was also performed for clinical reasons. Six specialists from the institutions listed above (three radiologists and three cardiologists) performed the ultrasound examinations, which measured intima-media thickness (IMT), the presence, type, and size of atherosclerotic plaques, as well as the blood flow rate and rate of narrowing in the ICA, external carotid artery (ECA), and CCA. The arithmetic mean of three measurements was used as the IMT for the CCA, ICA, and ECA. Data on general health, risk behaviors, and clinical, anthropometric, and laboratory parameters were collected, including age, gender, diastolic and systolic blood pressure, physical activity level, alcohol consumption, smoking status, waist circumference, BMI, glucose, glycated hemoglobin (HbA1c), total cholesterol, LDL, high-density lipoprotein (HDL), triglycerides, high-sensitivity C-reactive protein (hs-CRP), myocardial infarction (MI), coronary artery disease (CAD), duration of type 2 diabetes mellitus (T2DM), and arterial hypertension (AH).

### Ethical statements

All participants signed an informed consent form. The study was approved by the National Medical Ethics Committee (0120-316/2023/9) and was conducted in accordance with the Declaration of Helsinki.

### Genotyping

DNA was isolated from peripheral blood leukocytes using a QIAcube instrument (Qiagen GmbH, Hilden, Germany), following the V3 protocol. We used the commercial QIAamp DNA Blood Mini Kit (250) (Qiagen GmbH, Hilden, Germany), which included five reagents (AW1 buffer, AW2 buffer, AE buffer, AL buffer, and 96% ethanol) and protease (285 µL/200 µL blood). Genomic DNA (3–12 µg, 30–40 ng/µL) was isolated from 200 µL of blood as per the manufacturer’s instructions. The rs1333049 polymorphism was genotyped using the TaqMan SNP Genotyping Assay (Applied Biosystems, Foster City, CA, USA) according to the manufacturer’s instructions.

### Immunohistochemistry

We performed immunohistochemical analysis on 26 endarterectomy specimens, obtained from participants with advanced carotid atherosclerosis during the carotid endarterectomy procedure. The specimens were fixed in formalin and embedded in paraffin. Sections 5-µm thick were cut, deparaffinized, and rehydrated according to standard procedures. Anti-*CDKN2B* monoclonal antibodies (Thermo Fisher Scientific, Waltham, MA, USA) at a dilution of 1:40 were applied overnight at 4 ^∘^C. We used the Novo Link Max Polymer Detection System (Leica Biosystems, Newcastle upon Tyne, UK) to identify *CDKN2B*-positive and *CDKN2B*-negative cells. The numerical area density of *CDKN2B*-positive cells was calculated as the number of positive cells per square millimeter [17].

### Statistical analysis

Statistical analysis was performed using SPSS software (ver. 26.0, IBM SPSS, New York, NY, USA). The Shapiro–Wilk test was used to check the normality of data distribution. Continuous data with a normal distribution were presented as mean ± standard deviation (SD), while non-normally distributed data were presented as median and interquartile range. Categorical variables were reported as numbers and percentages. Normally distributed continuous data were compared using the unpaired Student *t*-test, and non-normally distributed data were compared using Mann–Whitney’s *U* test. The chi-square test was used to compare discrete variables. Logistic regression analysis was applied to variables that showed significant differences in univariate analysis (*P* < 0.05). The chi-square goodness of fit test was used to assess deviation from Hardy–Weinberg equilibrium (HWE).

**Table 1:** Clinical and laboratory characteristics of cases and controls

	**Case (*N* ═ 308)**	**Control (*N* ═ 573)**	***P* value**
Age (years)	70.94 ± 8.39	65.48 ± 11.20	***<0.001***
BMI (kg/m^2^)	28.02 ± 4.18	28.90 ± 4.21	***0.010***
Waist circumference (cm)	101.58 ± 12.25	97.28 ± 13.87	***<0.001***
SBP (mm Hg)	145.88 ± 20.49	147.93 ± 21.37	*0.23*
DBP (mm Hg)	80.36 ± 10.55	83.75 ± 10.61	***<0.001***
Fasting glucose (mmol/L)	6.77 ± 2.37	7.35 ± 2.75	***0.008***
Total cholesterol (mmol/L)	4.49 (3.70–5.30)	4.70 (4.00–5.60)	***<0.001***
HDL-cholesterol (mmol/L)	1.30 (1.10–1.50)	1.31 (1.00–1.50)	*0.71*
LDL-cholesterol (mmol/L)	2.40 (1.90–3.20)	2.70 (2.10–3.40)	***0.008***
Triglycerides (mmol/L)	1.40 (1.00–1.90)	1.40 (1.00–2.10)	*0.44*
HbA1c (%)	7.50 (7.05–9.03)	7.40 (6.62–8.20)	*0.078*
hs CRP (mg/L)	3.30 (2.90–7.00)	2.00 (1.00–3.98)	***<0.001***
Sex			***<0.001***
Male	209 (67.9%)	302 (52.7%)	
Female	99 (32.1%)	271 (47.3%)	
Smoking (%)			***<0.001***
Never + Former smoker	228 (74.0%)	507 (88.5%)	
Active smoker	80 (26.0%)	66 (11.5%)	
T2DM			***<0.001***
Yes	131 (42.5%)	348 (60.7%)	
No	177 (57.5%)	225 (39.3%)	

BMI: Body mass index; SBP: Systolic blood pressure; DBP: Diastolic blood pressure; HDL: High-density lipoprotein; LDL: Low-density lipoprotein; HbA1c: Glycated hemoglobin; hs CRP: High sensitivity C-reactive protein; T2DM: Type 2 diabetes mellitus.

**Table 2:** Distribution of rs1333049 polymorphism genotypes and alleles

***CDKN2B-AS1* rs1333049**	**Case (*N* ═ 308)**	**Control (*N* ═ 573)**	***P* value**
CC	71 (23.0%)	105 (18.3%)	***0.014***
CG	170 (55.2%)	293 (51.2%)	
GG	67 (21.8%)	175 (30.5%)	
Alleles			
C (MAF)	312 (50.6%)	503 (43.9%)	***0.007***
G	304 (49.4%)	643 (56.1%)	
HWE (*P* value)	0.0677	0.3607	
Dominant			
CC + CG	241 (78.2%)	398 (69.5%)	***0.005***
GG	67 (21.8%)	175 (30.5%)	
Recessive			
CC	71 (23.1%)	105 (18.3%)	*0.09*
CG + GG	237 (76.9%)	468 (81.7%)	

HWE: Hardy–Weinberg equilibrium.

**Table 3:** Logistic regression analysis adjusted for different variables according to genetic models

***CDKN2B-AS1* rs1333049**	**Count**	**OR (95% CI)**	***P value* for OR**
Codominant			
CC vs GG	71/105 vs 67/175	3.29 (1.32–8.91)	***0.014***
CG vs GG	170/293 vs. 67/175	2.50 (1.22–5.37)	***0.015***
Dominant			
[CC+CG] vs GG	241/398 vs 67/175	2.74 (1.36–5.71)	***0.006***
Recessive			
CC vs [CG+GG]	71/105 vs 237/468	1.60 (0.83–3.12)	*0.16*

Adjusted for: Age, BMI, waist circumference, DBP, fasting glucose, total cholesterol, LDL cholesterol, gender, smoking,T2DM. BMI: Body mass index; DBP: Diastolic blood pressure; LDL: Low-density lipoprotein; T2DM: Type 2 diabetes mellitus.

## Results

**Figure 1.**
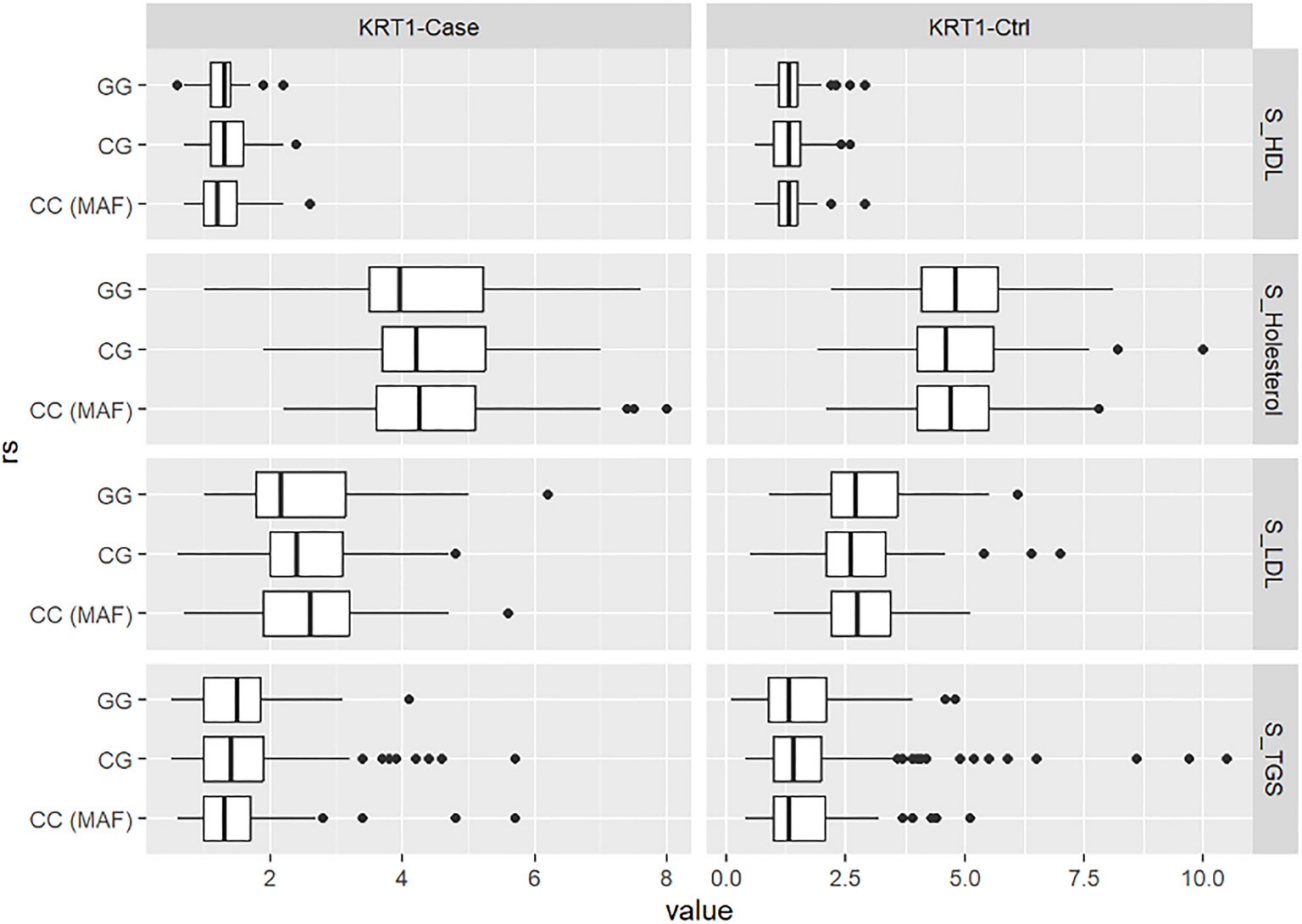
Results of the ANOVA analysis.

General information, medical history, anthropometric, and laboratory characteristics of the cases (participants with >75% obstruction of the ICA lumen) and controls (participants with hemodynamically insignificant stenosis of <50%) are shown in Table 1. The groups differed significantly in age, BMI, waist circumference, diastolic blood pressure (DBP), fasting glucose, total cholesterol, LDL, hs-CRP, sex, smoking history, and presence of T2DM. There was no statistically significant difference between the groups in systolic blood pressure, HDL cholesterol, triglycerides, or HbA1c. The case group was older, had lower BMI, DBP, fasting glucose, total cholesterol, and LDL cholesterol, but had a larger waist circumference and higher levels of hs-CRP. The proportion of smokers and T2DM patients was higher in the case group. The distribution of rs1333049 allele and genotype frequencies, as well as dominant and recessive inheritance models, are shown in Table 2. There was a statistically significant difference in the distribution of genotypes (*P* ═ 0.014) and alleles (*P* ═ 0.007) between the case and control groups. The genotype distribution did not significantly deviate from HWE. The dominant inheritance model showed a statistically significant association (*P* ═ 0.005), but the recessive model did not (*P* ═ 0.09). Logistic regression analysis was used to independently assess the association between rs1333049 and advanced carotid atherosclerosis after adjusting for age, BMI, waist circumference, DBP, fasting glucose, cholesterol, sex, smoking, and T2DM. The association was statistically significant under both the co-dominant (*P* ═ 0.014, OR ═ 3.29, 95% CI: 1.32–8.91, and *P* ═ 0.015, OR ═ 2.50, 95% CI: 1.22–5.37) and dominant (*P* ═ 0.006, OR ═ 2.74, 95% CI: 1.36–5.71) models (Table 3). We used ANOVA to examine whether rs1333049 genotypes were associated with total cholesterol, HDL, LDL, and triglyceride levels in the case and control groups separately (Figure 1). No statistically significant differences were observed between rs1333049 genotypes in either group.

**Figure 2.**
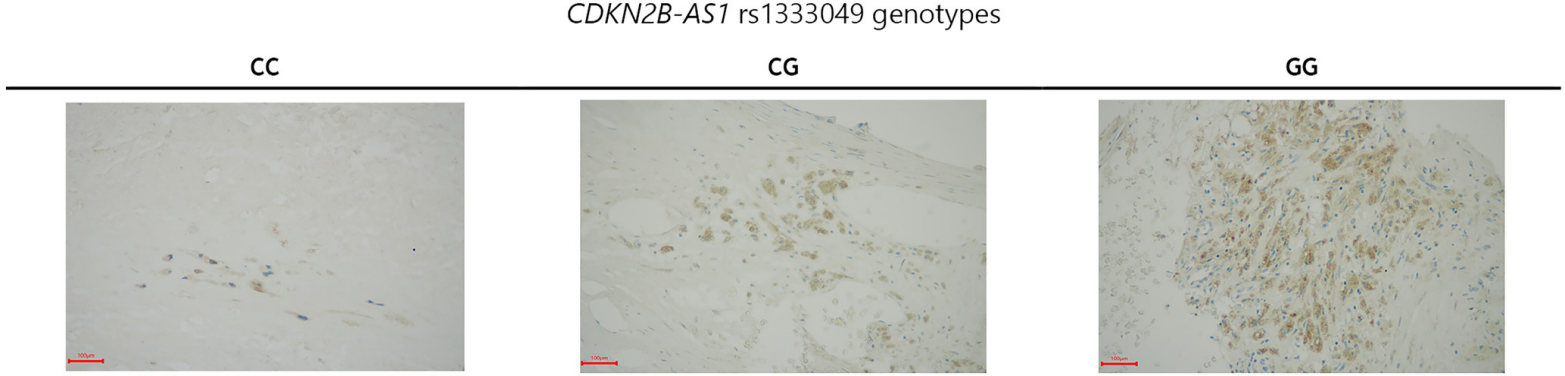
**Immunohistochemical staining of endarterectomy sequester samples relative to the *CDKN2B-AS1* rs1333049 genotypes.** The pictures were taken at 40× magnification. *CDKN2B*-positive cells are stained brown, *CDKN2B*-negative cells are not stained brown, only their nuclei are stained blue (The scale bar represents 100 µm).

We found a lower number of *CDKN2B*-positive cells in endarterectomy samples from participants with the CC genotype compared to those with the CG and GG genotypes (17 ± 15/mm^2^ vs 63 ± 32/mm^2^, *P* ═ 0.0012) (Figure 2).

## Discussion

Our study investigated the association between the rs1333049 SNP of *CDKN2B-AS1* and advanced carotid artery atherosclerosis in the Slovenian Caucasian population. Logistic regression analysis showed an association between rs1333049 and advanced carotid artery atherosclerosis in two inheritance models. The CC and CG genotypes were associated with a higher risk of advanced atherosclerosis than the GG genotype, according to the co-dominant and dominant inheritance models. This is the first study to investigate the association between rs1333049 and advanced carotid atherosclerosis (>75% ICA lumen obstruction). According to the dominant inheritance model, participants with the CC genotype had a 3.29-fold higher risk of advanced carotid atherosclerosis than those with the GG genotype, and participants with the CG genotype had a 2.5-fold higher risk. In the co-dominant model, the combined CC and CG genotypes increased the risk by 2.74 times compared to the GG genotype. Two other studies have investigated this association in Caucasian populations. Plichart et al., using data from the Three Cities Study and the Vascular Aging Study (two French cohorts with a combined sample size of 4097), reported an association between each copy of the C allele and carotid plaque presence [18]. In the Bruneck study, the authors found an association between the C allele and both atherosclerosis progression and carotid stenosis of more than 40% [19]. In the Han Chinese population, Lin et al. [20] reported that the C allele was a risk factor for higher ICA IMT and plaque presence. CC and CG genotypes were more likely to have carotid plaque than the GG genotype. These findings align with our study, showing that the C allele is associated with carotid atherosclerosis in both Caucasian and Han Chinese populations. Interestingly, cases had lower total cholesterol and LDL cholesterol than controls, likely due to better therapy adherence in the case group. Some studies have suggested an association between the C allele of rs1333049 and lipid levels (e.g., total cholesterol and triglycerides), suggesting this may increase cardiovascular risk. We used ANOVA to explore whether rs133304 genotypes were associated with lipid status but found no statistically significant associations in either group. Similarly, Plichart et al. [18] and Ye et al. [19] found no associations between rs1333049 genotypes and lipid levels. However, a meta-analysis by Wei et al. [21] found a significant association between the C allele and total cholesterol and triglycerides in CAD patients. We observed a statistically significant difference in the number of *CDKN2B*-positive cells in endarterectomy samples between rs1333049 genotypes. Carriers of the C allele had fewer *CDKN2B*-positive cells. Motterle et al. also reported lower *CDKN2B* and *CDKN2A* expression in CC genotype carriers compared to GG carriers, though the difference was not statistically significant for p16/INK4a. They also found an association between the CC genotype and increased VSMC content in plaques [15]. The clinical manifestations of carotid atherosclerosis include transient ischemic attacks (TIAs) and ISs [22]. In 2017, the global incidence of IS was 101.3 per 100,000 people, and in the EU, it was 219.4 per 100,000 [23, 24]. While 87% of all strokes globally are IS, in Slovenia, 60.60% of strokes in 2019 were IS [25, 26]. Risk factors for atherosclerosis include smoking, physical inactivity, metabolic syndrome, dyslipidemia, hypertension, and genetic/epigenetic factors [3, 25]. Previous studies have also linked rs1333049 to carotid artery calcification in both Caucasian and Chinese populations. For example, Bos et al. [27] found an association between rs1333049 and carotid artery calcification in the Rotterdam Study, independent of other cardiovascular risk factors. Zhang et al. observed a similar association in a cohort of 878 Chinese IS patients. However, a meta-analysis of the two studies found no significant association between rs1333049 and carotid calcification [28]. The CC genotype of rs1333049 is associated with increased angiogenesis, but also with inhibition of neovessel maturation in the atherosclerotic plaque [29]. In terms of CAD, several studies have examined rs1333049. Huang et al. found an association between rs1333049 and CAD but not with MI or stroke. This association was significant in East Asians but not in Caucasians [6]. Ozuynuk-Ertugrul et al. [30] found no association between rs1333049 and CAD in Turkish participants, but reported a link between the GG genotype and diabetes mellitus (DM) in males. In Slovenian patients with T2DM, Tibaut et al. [31] reported an association between the CC genotype and MI. Borghini et al. [32] also found an association between the C allele and major adverse cardiac events (MACE) in Italian patients with stable CAD, as well as with shorter leukocyte telomere length. These findings support the idea that rs1333049 is associated with atherosclerosis, regardless of its specific clinical manifestation. Several studies have also investigated the rs1333049 SNP in the context of chronic inflammatory diseases, such as rheumatoid arthritis (RA), psoriasis, psoriatic arthritis, and periodontitis, which increase cardiovascular risk [33, 34]. Genetic studies that include patients with these conditions could enhance our understanding of atherosclerosis pathogenesis. García-Bermúdez et al. [35] found no association between cardiovascular disease in RA patients and rs10116277 and rs1537375, two SNPs in *CDKN2B-AS1*. López-Mejías et al. reported an association between subclinical atherosclerosis and the gene for retinoic acid receptor beta (RARB) but did not find an association with rs1333049 [36]. These results highlight the complex genetic mechanisms underlying atherosclerosis. In the future, our findings may be used in the context of preventive medicine. The rs1333049 polymorphism could be one of the SNPs in a selection of genetic markers. Patients with early stages of carotid atherosclerosis could be screened using this selection and then the patients with the CC or CG genotype would have either more frequent check-ups or a more intensive treatment plan prescribed in order to address their increased risk for developing advanced atherosclerosis. However, we must address some limitations of our study. Our study had a relatively small sample size, which may affect the validity of our findings. The power of our study was 0.82 which suggests that our sample size was not too small. All participants were Caucasians and, therefore, our results are only applicable to a Caucasian population. We did not investigate the role of other SNPs that may influence carotid atherosclerosis, which may have affected our results. Also, we only performed immunohistochemical analysis on endarterectomy samples from 26 participants because we did not have more material. The association between the rs1333049 polymorphism and the expression of *CDKN2B* should be validated on a larger sample.

## Conclusion

In conclusion, the C allele of rs1333049 in the *CDKN2B-AS1* gene may be a risk allele for advanced carotid atherosclerosis in the Slovenian Caucasian population.

## Data Availability

The data presented in this study are available on request from the corresponding author.

## References

[1] Libby P (2021). Inflammation during the life cycle of the atherosclerotic plaque. Cardiovasc Res.

[2] Björkegren JLM, Lusis AJ (2022). Atherosclerosis: recent developments. Cell.

[3] Fan J, Watanabe T (2022). Atherosclerosis: known and unknown. Pathol Int.

[4] Tcheandjieu C, Zhu X, Hilliard AT, Clarke SL, Napolioni V, Ma S (2022). Large-scale genome-wide association study of coronary artery disease in genetically diverse populations. Nat Med [Internet].

[5] Munir MS, Wang Z, Alahdab F, Steffen MW, Erwin PJ, Kullo IJ (2014). The association of 9p21-3 locus with coronary atherosclerosis: A systematic review and meta-analysis. BMC Med Genet.

[6] Huang Y, Jin H, Yang G (2020). Associations between common polymorphisms of *CDKN2B*-AS and susceptibility to ASCVD. Angiology.

[7] Shima K, Nosho K, Baba Y, Cantor M, Meyerhardt JA, Giovannucci EL (2011). Prognostic significance of *CDKN2A* (p16) promoter methylation and loss of expression in 902 colorectal cancers: cohort study and literature review. Int J Cancer J Int Cancer.

[8] Jang W, Park J, Kwon A, Choi H, Kim J, Lee GD (2019). *CDKN2B* downregulation and other genetic characteristics in T-acute lymphoblastic leukemia. Exp Mol Med.

[9] De Braekeleer M, Douet-Guilbert N, De Braekeleer E (2017). Prognostic impact of p15 gene aberrations in acute leukemia. Leuk Lymphoma.

[10] Ren W-H, Li Y-W, Li R, Feng H-B, Wu J-L, Wang H-R (2015). P15 gene methylation in hepatocellular carcinomas: a systematic review and meta-analysis. Int J Clin Exp Med [Internet].

[11] Serra S, Chetty R (2018). p16. J Clin Pathol.

[12] Kojima Y, Downing K, Kundu R, Miller C, Dewey F, Lancero H (2014). Cyclin-dependent kinase inhibitor 2B regulates efferocytosis and atherosclerosis. J Clin Invest.

[13] VinuÉ Á, MartÍnez-HervÁs S, Herrero-Cervera A, SÁnchez-GarcÍa V, AndrÉs-Blasco I, Piqueras L (2019). Changes in *CDKN2A/2B* expression associate with T-cell phenotype modulation in atherosclerosis and type 2 diabetes mellitus. Transl Res.

[14] Kuo C-L, Murphy AJ, Sayers S, Li R, Yvan-Charvet L, Davis JZ (2011). *CDKN2A* is an atherosclerosis modifier locus that regulates monocyte/macrophage proliferation. Arterioscler Thromb Vasc Biol.

[15] Motterle A, Pu X, Wood H, Xiao Q, Gor S, Liang Ng F (2012). Functional analyses of coronary artery disease associated variation on chromosome 9p21 in vascular smooth muscle cells. Hum Mol Genet.

[16] Bai N, Liu W, Xiang T, Zhou Q, Pu J, Zhao J (2022). Genetic association of ANRIL with susceptibility to Ischemic stroke: a comprehensive meta-analysis. PLoS One.

[17] Petrovič D, Letonja J, Petrovič D (2024). SMAD3 rs17228212 polymorphism is associated with advanced carotid atherosclerosis in a Slovenian population. Biomedicines.

[18] Plichart M, Empana J-P, Lambert J-C, Amouyel P, Tiret L, Letenneur L (2012). Single polymorphism nucleotide rs1333049 on chromosome 9p21 is associated with carotid plaques but not with common carotid intima-media thickness in older adults. A combined analysis of the Three-City and the EVA studies. Atherosclerosis.

[19] Ye S, Willeit J, Kronenberg F, Xu Q, Kiechl S (2008). Association of genetic variation on chromosome 9p21 with susceptibility and progression of atherosclerosis: a population-based, prospective study. J Am Coll Cardiol.

[20] Lin H-F, Tsai P-C, Lin R-T, Khor G-T, Sheu S-H, Juo S-HH (2010). Sex differential genetic effect of chromosome 9p21 on subclinical atherosclerosis. PloS One.

[21] Wei B, Liu Y, Li H, Peng Y, Luo Z (2022 [cited 2023 Nov 21]). Effect of 9p21.3 (lncRNA and *CDKN2A/2B*) variant on lipid profile. Front Cardiovasc Med [Internet].

[22] Arasu R, Arasu A, Muller J (2021 [cited 2023 Oct 10]). Carotid artery stenosis: an approach to its diagnosis and management. Aust J Gen Pract [Internet].

[23] Saini V, Guada L, Yavagal DR (2021). Global epidemiology of stroke and access to acute ischemic stroke interventions. Neurology.

[24] Wafa HA, Wolfe CDA, Emmett E, Roth GA, Johnson CO, Wang Y (2020). Burden of stroke in Europe. Stroke.

[25] Benjamin EJ, Muntner P, Alonso A, Bittencourt MS, Callaway CW, Carson AP (2019). Heart disease and stroke statistics—2019 update: a report from the American Heart Association. Circulation.

[26] Dokova KG, Feigin VL (2022). Trends in stroke burden in central and Eastern Europe from 1990–2019. Neuroepidemiology.

[27] Bos D, Ikram MA, Isaacs A, Verhaaren BFJ, Hofman A, van Duijn CM (2013). Genetic Loci for coronary calcification and serum lipids relate to aortic and carotid calcification. Circ Cardiovasc Genet.

[28] Zhang Y, Wang L, Zhang Z, Zhang Z, Zhou S, Cao L (2015). Shared and discrepant susceptibility for carotid artery and aortic arch calcification: a genetic association study. Atherosclerosis.

[29] Nanda V, Downing KP, Ye J, Xiao S, Kojima Y, Spin JM (2016). *CDKN2B* Regulates TGFβ signaling and smooth muscle cell investment of hypoxic neovessels. Circ Res.

[30] Ozuynuk-Ertugrul AS, Kirsan CB, Erkan AF, Ekici B, Komurcu-Bayrak E, Coban N (2024). Genetic variants of ANRIL and coronary artery disease: insights from a Turkish study population. Gene.

[31] Tibaut M, Naji F, Petrovič D (2022). Association of myocardial infarction with *CDKN2B* Antisense RNA 1 (*CDKN2B-AS1*) rs1333049 polymorphism in slovenian subjects with Type 2 diabetes mellitus. Genes (Basel).

[32] Borghini A, Mercuri A, Campolo J, Parolini M, Ndreu R, Turch S (2023). Influence of Chromosome 9p21.3 rs1333049 variant on telomere length and their interactive impact on the prognosis of coronary artery disease. J Cardiovasc Dev Dis.

[33] Visseren FLJ, Mach F, Smulders YM, Carballo D, Koskinas KC, Back M (2021). 2021 ESC guidelines on cardiovascular disease prevention in clinical practice: developed by the task force for cardiovascular disease prevention in clinical practice with representatives of the European Society of Cardiology and 12 medical societies with the special contribution of the European Association of Preventive Cardiology (EAPC). Eur Heart J.

[34] Castañeda S, Nurmohamed MT, González-Gay MA (2016). Cardiovascular disease in inflammatory rheumatic diseases. Best Pract Res Clin Rheumatol.

[35] García-Bermúdez M, López-Mejías R, Genre F, Castañeda S, González-Juanatey C, Llorca J (2013). Single-nucleotide polymorphisms at the 9p21.3 genomic region not associated with the risk of cardiovascular disease in patients with rheumatoid arthritis. Tissue Antigens.

[36] López-Mejías R, Carmona FD, Genre F, Remuzgo-Martínez S, González-Juanatey C, Corrales A (2019). Identification of a 3’-untranslated genetic variant of RARB associated with carotid intima-media thickness in rheumatoid arthritis: a genome-wide association study. Arthritis Rheumatol.

